# Virtual Reality for Analgesia During Intrauterine Device Insertion: Randomized Controlled Trial

**DOI:** 10.2196/72917

**Published:** 2025-09-08

**Authors:** Chloe Higgins, Claudia Zecena Morales, Judi Hocking, Kate Tyson, Cheryl Leung, Luke Larmour, Paul Leong, Beverley Vollenhoven

**Affiliations:** 1 Women's and Newborn Program, Monash Health, 246 Clayton Rd, Melbourne, 3168, Australia, 61 395946666; 2 Department of Obstetrics and Gynaecology, Monash University, Melbourne, Australia; 3 Department of Lung and Sleep, Monash Health, Melbourne, Australia; 4 Department of Respiratory Research, Monash University, Melbourne, Australia

**Keywords:** virtual reality, intrauterine device, pain, anxiety, contraception

## Abstract

**Background:**

Intrauterine devices (IUDs) are safe and effective long-acting reversible contraceptive therapies that are also used as minimally invasive treatment for heavy menstrual bleeding, endometrial hyperplasia, and early-stage endometrial cancer. Despite many advantages, IUDs are underused predominantly due to patient discomfort. Although many techniques have been explored previously in the literature, there is currently little consensus on effective analgesic strategies. Virtual reality (VR) has demonstrated moderate benefits in acute pain management and has been explored for outpatient hysteroscopy.

**Objective:**

This study aims to explore the effectiveness of VR in improving patient pain and anxiety during outpatient IUD insertion.

**Methods:**

This randomized controlled trial compared the use of a VR headset to standard care during IUD insertion in the outpatient clinic setting. VR content was delivered via smartphone and headset, providing patients with a relaxing 3D video environment. Outcomes measured were patient-reported pain and anxiety, as well as satisfaction reported using a questionnaire. Secondary outcomes included clinician-reported ease of insertion and time required to complete the procedure.

**Results:**

A total of 70 patients were recruited, with 34 randomized to the control group and 36 randomized to VR headset use. Patients with VR headsets reported a mean pain score of 5.5 (SD 3.2) during IUD insertion, which was not significantly different to 4.3 (SD 3.2) for the control group (*P*=.15). Mean anxiety scores during the procedure were 4 (SD 3) in the VR group, compared to 4.8 (SD 3.5) in the control group, which was also not significantly different (*P*=.37). Anxiety was the most significant predictor of pain, and this, in turn, significantly increased insertion time (*P*<.001). Among patients who responded to and benefitted from VR use, baseline anxiety was significantly lower than in those who did not (*P*<.001). Satisfaction with the use of VR headsets was overall high, and recommendation scores for the use of VR headsets were also high. There were no significant adverse effects experienced with the use of the intervention, with only 1 patient reporting nausea after IUD insertion.

**Conclusions:**

The use of VR headsets did not significantly alter the pain or anxiety experienced by patients during IUD insertion; however, satisfaction and recommendation that others use VR were high, which may suggest other benefits to their use. In addition, preprocedural anxiety appears to have a significant adverse impact on pain scores and the ability of patients to benefit from the VR headsets. This is an important contribution to the previously ambiguous data regarding VR use for gynecological procedures and highlights a new avenue for improving the patient experience.

## Introduction

Intrauterine devices (IUDs) are extremely effective long-acting reversible contraceptives that have been shown to be safe and convenient [1-4]. IUDs containing levonorgestrel are used as minimally invasive and fertility-preserving management options for gynecological conditions, including heavy menstrual bleeding, endometrial hyperplasia, and early-stage endometrial cancer [5]. Despite being the most used reversible contraceptive method globally and having substantial potential benefits, IUDs continue to be underused in many settings, including high-income countries [4,6]. Several barriers to IUD use have been hypothesized, and a major factor is the procedural experience, particularly providing accessible and acceptable insertion for patients [2,4].

Gynecological procedures, including IUD insertions, are increasingly being undertaken in the outpatient clinic setting as this is cheaper, more convenient, and avoids the risks of procedural sedation [7]. Despite being more accessible, IUD in this setting is associated with patient discomfort and anxiety, with pain scores usually ranging from 5 to 7 out of 10, which is a significant barrier to patient acceptability [2,8,9]. IUDs are technically feasible to insert for most outpatients, including nulligravida and patients with previous cesarean section [10]; however, these are common predictors of IUD insertion pain, in addition to remoteness from last pregnancy, dysmenorrhea, anticipation, and IUD type used (levonorgestrel being more painful than copper) [3,11].

IUD insertion can be undertaken in the operating theater under general anesthesia or procedural sedation. While this does alleviate patient discomfort, operating theater costs and access make this prohibitive for routine practice [12]. Compounding these reasons, the difficulty of access to operating theaters during the pandemic, and the constant requirement to keep operating theater time as training opportunities for junior doctors, the incentive to optimize the experience of outpatient insertion of IUDs is as strong as ever [12].

There is currently no consensus regarding the analgesic of choice during outpatient IUD insertion. Previously studied methods include oral or intravenous sedation, local anesthetic topically or as a cervical block, oral analgesics, and distraction techniques [7]. Common techniques such as nonsteroidal anti-inflammatory drugs, misoprostol, local anesthetic gel, and intrauterine instillation of local anesthetic have been found to be ineffective [1-3,6,13]. Paracervical local anesthetic injections have been suggested to be of some benefit during insertion; however, the injections themselves can be painful [1]. Tramadol and naproxen have shown possible benefits in small numbers in studies exclusively investigating nulliparous women; however, this was not replicated in follow-up studies [2,11,13]. Further options need to be explored to improve acceptability and patient uptake.

Virtual reality (VR) is gaining momentum as a novel nonpharmacological pain relief option, and its potential role in health care is increasingly relevant, as technological developments have improved its accessibility [14]. VR creates an immersive virtual environment for the user [7]. It can be delivered using a range of hardware, most commonly in the form of a head-mounted display with built-in headphones. It can present as an active VR, where participants interact with the content, such as in games, or passive virtual environments, such as movies. It has been suggested that active VR may have stronger analgesic properties than passive [14].

VR has demonstrated moderate benefits in acute pain management, including burns, labor, dressing changes, and periodontal procedures [15]. It is theorized that VR provides distraction from pain by multisensorial stimulation, perhaps via the gated-pain theory [7,14]. Reported side effects of VR are infrequent, mild, and self-limiting. They include nausea, vomiting, and eyestrain, collectively referred to as “cybersickness” [14]. If any of these events occur, the headset can be removed with rapid resolution of symptoms.

The possible benefits of VR in pain and periprocedural anxiety scores, combined with its minimal risks to patients, highlight the potential of this method and hence the need for further exploration of its use and cost-effectiveness in various settings [7,14,15]. Despite its initial promising results, particularly in pediatric care, it has been hypothesized that the effectiveness of VR is not necessarily generalizable to adult populations and other procedures [15]. The benefits likely vary significantly between patient populations and indications for use, such that studies in specific settings are recommended [14]. It has therefore been suggested for consideration for IUD insertion, with the hypothesis that it may provide an effective distraction technique to help with alleviating pain and anxiety, thereby improving patient access to outpatient IUD insertion [7,16,17].

A search of the literature for VR use in IUD insertion yielded only 2 research trials. One randomized controlled study performed in France with 50 patients per group found no change in perceived pain in women using VR therapy compared to those without, but rather noted the strongest predictor of pain being preprocedure anxiety [16]. The second study performed in Turkey had directly contradicting results, demonstrating an improvement in pain, anxiety, and satisfaction scores in those using a VR headset compared to the control group, with 40 patients in each group [17]. Research performed on other gynecological procedures, such as outpatient hysteroscopy, disagrees on whether pain scores are improved but shows promising improvements in anxiety and patient satisfaction associated with the use of VR [7,18,19].

The objective of this study was therefore to explore the impact of VR on patient-reported pain during IUD insertion in a tertiary gynecology hospital setting in Australia. Secondary aims included the effect of VR on patient anxiety and ease of IUD insertion, while also assessing the feasibility of introducing this as an analgesic method in the outpatient gynecological setting.

## Methods

### Recruitment

In this single-center randomized controlled trial, patients attending for IUD insertion in the outpatient gynecology clinic were screened for eligibility. Patients were eligible if aged 18 years and older and able to provide written informed consent. Patients were excluded if they had contraindications to IUD insertion, a history of epilepsy or vestibular disorders contraindicating VR use, an inability to consent, or if they had taken prescription pain relief prior to the procedure (simple analgesia was permitted).

Patients were randomized on the day of IUD insertion in the outpatient clinic to either the intervention group (addition of VR to standard care) or the control group (standard care). A computerized random sequence was generated, with allocations then placed in sequentially numbered opaque envelopes by a blinded third party. These envelopes were opened only after participants were enrolled in the study by the researcher, who was not otherwise involved in the patient’s care. Participants were not paid for their participation. Given the obvious nature of VR as an intervention and the subjective nature of pain and anxiety as outcomes, blinding was not possible.

**Figure 1.**
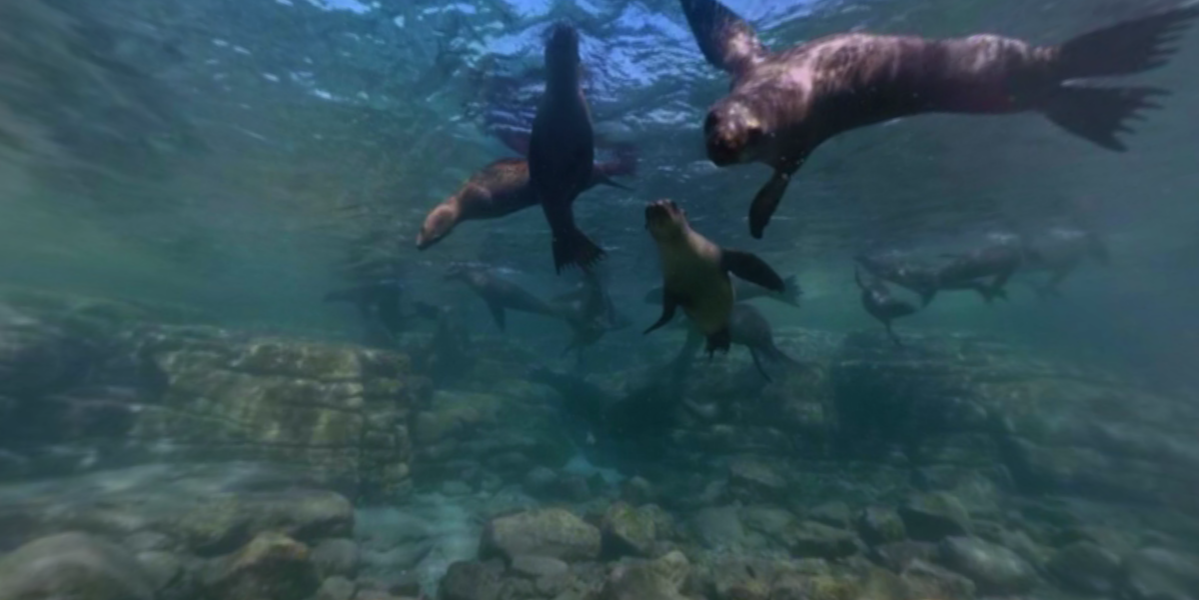
Still image captured from virtual reality seals swimming.

VR digital content was delivered via smartphone and headset during the IUD insertion procedure. The content provided a relaxing, noninteractive 3D video of seals swimming with gentle classical music (Figure 1). The visual content was chosen by the investigators to ensure its suitability for the patient population and minimize any patient reactions to objectionable content. Content containing dialogue was considered inappropriate as it would interfere with clinical communication during this intimate procedure. Standard care included occasional use of a lignocaine spray to the cervix according to the clinician’s preference, and this was therefore recorded as part of the data collection.

Several pain scales exist and can be used to quantify the pain intensity from IUD insertion, including the Visual Analog Scale (VAS), the Verbal Rating Scale, and the Numerical Rating Scale [20]. The VAS is reliable, valid, and sensitive to subtle differences and changes in pain intensity [20]. For this reason, it is the most consistently used tool [21] and has been used in prior studies of discomfort associated with IUD insertion [3,5]. VAS differences of 10 mm are considered clinically significant from previous studies [22,23].

Patient demographic information was collected by reviewing the patient’s medical records and full consultation notes. In addition, patients were asked to plot their anxiety prior to the procedure, and their pain and anxiety during the procedure on a linear 10-cm VAS. Numerical VAS scores were then obtained by measuring the distance along the line to the nearest millimeter. Patients also completed a questionnaire on their satisfaction, recommendations, and side effects after the procedure. The time required for IUD insertion, clinician-reported ease of insertion on a scale of 1 to 5, and number of attempts required for successful IUD insertion (defined as the number of attempts to pass the IUD through the cervix) were also reported (Multimedia Appendix 1).

### Statistical Analysis

Statistical analyses were conducted in SPSS 25 (IBM Corp). Significance was defined as *P* <.05. Two-tailed *t*-tests were performed for group-wide comparisons.

### Power Calculations

For our power calculation, we assumed an equal SD of 14 mm in VAS pain scores between groups based on previous studies of VAS pain scores in women with pelvic pain during IUD insertion [24]. To achieve 80% of detecting a 10-mm difference between groups with a type 1 error rate of 0.05, we required a sample size of 31 in each group. Accounting for a compliance rate of 90%, we aimed for a sample size of 70 participants.

### Ethical Considerations

The study was approved by Monash Health Ethics Committee, Clayton, Victoria, Australia (ethics approval reference number 80700 and Monash Health reference number RES-21-0000-690A). The trial was prospectively registered (Australia and New Zealand Clinical Trial Registry ACTRN12622000088741p). Written informed consent was obtained from all participants following counseling, with time allowed for patients to review the participant information and consent form. Patients were able to opt out of the study at any stage if they chose to withdraw consent to participate. There were no payments or incentives or compensation provided that could result in pressure to participate. Data collected were deidentified and stored on secure password-protected servers, with all data converted to electronic copies and hard copies destroyed in confidential waste to protect patient privacy.

## Results

**Figure 2.**
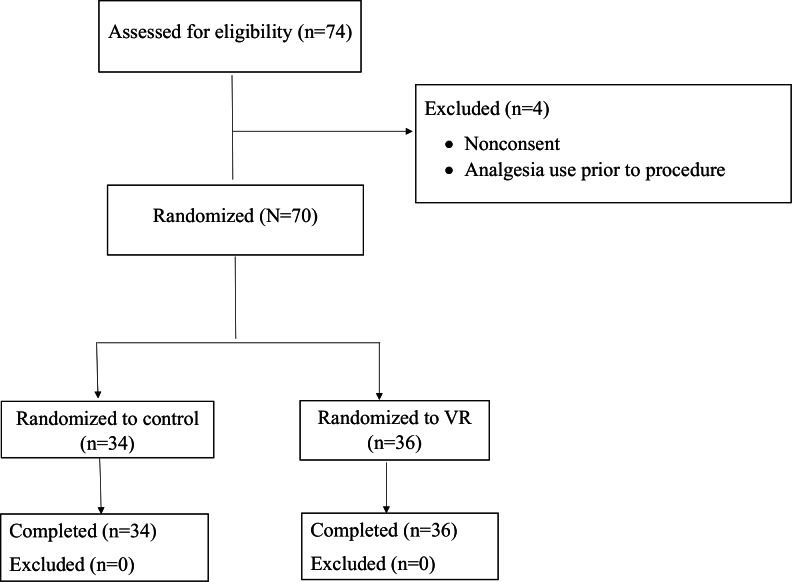
CONSORT (Consolidated Standards of Reporting Trials) diagram of participant inclusion and exclusion. VR: virtual reality.

Participant flow is described in Figure 2.

Of the 70 patients enrolled in the study, there were 34 in the control group and 36 in the VR intervention group. Their baseline characteristics are outlined in Table 1. Of the 34 control group participants, 31 had levonorgestrel-containing devices inserted, and of the 36 VR intervention group participants, 34 had levonorgestrel-containing devices. The remaining participants had copper IUDs.

Of note, despite randomization, there was numerical imbalance with patients in the VR group being more likely to have previously had an IUD, previously used a VR headset, and a history of chronic pain.

There was no significant difference in intraprocedural pain scores with the use of VR headsets (Table 2). Similarly, preprocedural and intraprocedural anxiety scores did not significantly differ (Table 2).

The use of VR headsets also did not appear to have a significant impact on the IUD insertion process as clinician-reported ease of insertion, time to insertion, and the number of insertion attempts did not differ (Table 3).

Satisfaction with the use of VR headsets was overall high (mean 7.3 out of 10, SD 2.99), and recommendation scores for use of VR headsets were also high (mean 8.35 out of 10, SD 2.24). Satisfaction and recommendation were highly correlated, and a high satisfaction score was weakly associated with lower intraprocedural anxiety scores (*r*=0.33, *P*=.06).

Preprocedural anxiety was significantly correlated with intraprocedural anxiety (*P*<.001). Overall, anxiety was the most significant predictor of pain during insertion (*P*=.001). Higher pain was also a significant predictor of increased procedural time (*P*<.001).

Subgroup analysis was performed to isolate the patients who most benefit from VR. Responders to VR were defined as patients in the VR intervention group who had pain scores of less than or equal to 5. This score was chosen due to literature findings of pain scores of 5‐7 out of 10 for IUD insertion with placebo use [8]. Patients who were classified as responders were compared with nonresponders (those with pain scores greater than 5 in the VR intervention group). The characteristics of these 2 groups were markedly different, with significantly lower preprocedural and intraprocedural anxiety in the responders than nonresponders (*P*<.05). Baseline characteristics, including BMI, analgesic spray, prior IUD insertion, and procedure success, were not significantly different between the 2 subgroups.

There were no significant adverse effects experienced with the use of the intervention, with only 1 patient reporting nausea after IUD insertion.

**Table 1.** Baseline population characteristics.

Characteristics	Control (n=34)	VR^b^ (n=36)
Age (years), mean (SD)	34 (6)	36 (7)
BMI (kg/m^2^), mean (SD)	30 (8)	31 (7)
Gravida, n	2	2
Parity, n	2	2
Previous vaginal deliveries, mean (SD)	1 (1)	1 (1)
Time since last vaginal birth in years, median (IQR)	1.5 (0.21‐3.25)	0.58 (0.19‐4)
Previous cervical instrumentation, n (%)	11 (32)	5 (14)
Anatomical distortions, n (%)	6 (18)	6 (17)
Prior IUD^a^, n (%)	10 (29)	15 (42)
Previous VR^b^ use, n (%)	3 (9)	11 (30)
Use of analgesic spray, n (%)	11 (32)	10 (28)
Indication for IUD^a^, n (%)		
Contraception	32 (94)	27 (75)
Abnormal bleeding	0 (0)	5 (14)
Both	2 (6)	2 (6)
Mental health history, n (%)	5 (15)	6 (17)
Chronic pain history, n (%)	0 (0)	5 (14)

a IUD: intrauterine device.

b VR: virtual reality.

**Table 2.** Patient pain and anxiety outcomes.

Characteristics	Control (n=34)	VR^a^ (n=36)	*P* value
Pain score, mean (SD)	4.3 (3.2)	5.5 (3.2)	.15
Anxiety prior to procedure, mean (SD)	4.2 (2.8)	4.6 (3.1)	.56
Anxiety during procedure, mean (SD)	4.8 (3.5)	4 (3)	.37

a VR: virtual reality.

**Table 3.** Effect of virtual reality use on intrauterine device insertion.

Characteristics	Control (n=34)	VR^a^ (n=36)	*P* value
Number of attempts to successfully insert IUD^b^, mean (SD)	1.09 (0.59)	0.94 (0.65)	.73
Clinician reported ease of insertion (1-5), mean (SD)	2.75 (1.48)	2.75 (1.63)	.41
Time for insertion (minutes:seconds), mean (SD)	12:06 (0:04)	12:06 (0:02)	.61

a VR: virtual reality.

b IUD: intrauterine device.

## Discussion

### Overview

Despite the many benefits of IUDs, patient discomfort is a significant barrier to accessing outpatient insertion [2,4]. There is little evidence and no clear consensus on the best methods of addressing this discomfort without this being performed under general anesthesia, which itself presents costs and risks [12].

### Principal Findings

This study found no significant difference in anxiety or pain with VR headset use and identified anxiety as the most significant predictor of pain.

Interestingly, despite pain and anxiety scores not significantly differing between the 2 groups, the VR headsets may have benefits that may still be relevant for procedural acceptability. The VR intervention was effective and well-received in a subpopulation of responders, particularly those with reduced levels of pre-existing anxiety. Satisfaction and recommendation for the VR headsets were overall very high, demonstrating patient appreciation of these devices. Patients were provided with the opportunity to comment on their experience, and those who did were overwhelmingly positive. The reasons for high satisfaction and recommendation for VR use appear to be related predominantly to the distraction effect.

Patients’ comments particularly highlighted that they appreciated the distraction provided by the headsets, with 1 patient reporting the distraction was helpful for anxiety, while others stated the distraction helped them to forget about the procedure. Other possible reasons for the positive patient feedback for the VR devices may include the novelty of the equipment or other psychological benefits, which, although difficult to quantify, may improve the acceptability of outpatient IUD insertion with VR use despite the lack of improvement in pain scores.

### Comparison to Prior Work

As VR grows in its implementation in the health care space, several studies have recently been published on its use in outpatient gynecological procedures. The existing body of evidence relating directly to IUD insertion is scant and contradictory, with the study by Benazzouz et al directly contradicting findings from Oz et al [16,17]. Both studies were randomized trials, recruiting patients in a contraceptive clinic on the day of IUD insertion, applying VR headsets, and collecting similar data measures, including the use of the VAS for pain rating; however, the studies had important methodological differences.

The study by Benazzouz et al [16] was conducted in France in 100 patients with a mean age of roughly 24 years and found no difference in pain or anxiety with VR headset use, but preprocedure anxiety was identified as a significant predictor of pain. Postprocedure mean pain scores in this study were around 5, similar to our study. Most patients underwent copper IUD insertion, which is known to be less painful, and approximately 80% of patients took preprocedural analgesia that included opiates and other prescription medications, factors which may have biased results toward the null.

The study by Oz et al [17] was conducted in Turkey and recruited 80 patients with a mean age of around 24 years. They used copper IUDs and found a reduction in postprocedural pain and anxiety scores with the use of VR. Mean pain scores were around 2 cm. This study’s strengths included its uniform use of only 1 VR visual and audio immersion option for all participants, similar to our study. However, the description of inclusion criteria was limited, and confounders such as previous cervical instrumentation, chronic pain, or other concurrent analgesia use were not stated.

Our study is the first to evaluate the use of VR for IUD insertion in an Australian setting. It has multiple similarities to the study by Benazzouz et al [16], including similar mean pain scores and no benefit for pain or anxiety with VR device use. Its prospective design and randomization are strengths and add to the sparse body of literature. We enrolled a representative sample of individuals who would be seen for IUDs in an Australian tertiary hospital context. The IUDs used in our study were predominantly levonorgestrel devices, in contradistinction to the other studies. Other strengths include practical, real-life, broad inclusion criteria, and we excluded people who had taken pain relief other than simple analgesia.

### Limitations

This study has limitations. First, although our study was practically oriented and therefore purposefully limited in the data instruments collected, this limited our ability to more deeply understand which subpopulations might benefit from this intervention or not. Deeper characterization of responder and nonresponder populations, particularly with regard to anxiety, would be an important consideration for future studies.

Second, this was a single-center study with multiple operators, so results may not apply to other operators or populations; however, the outcomes observed are plausible and concordant with other data. It is also possible that factors inherent to our study population may have limited the efficacy of VR headset use.

Third, although randomization is supposed to produce confounder balance, there were apparent differences between groups in our study. The VR group was more likely to have IUDs inserted for abnormal uterine bleeding and a history of chronic pain and less likely to have undergone previous cervical instrumentation. These factors could contribute to the patients’ interactions with pain experiences and expectations prior to IUD insertion, which in turn can worsen pain. These confounders may have blunted the measured effect in this study. It is also possible that other potential unmeasured confounders may have impacted the outcome (eg, unmeasured history of substance use).

In addition, despite a formal power calculation, we cannot exclude the possibility that this study was underpowered, given that we observed a much higher SD in pain scores than we had anticipated. Pain is an inherently subjective phenomenon, and individual variability in the reporting of these outcomes can be vexing. If future studies are to be performed, these could be on a larger scale in a multicenter context to explore a broader patient population and improve the generalizability of results while reducing the likely impact of possible confounders.

Another possible limiting factor could be that the type of content provided to patients, though thought to be appropriate, may not have been optimal. By standardizing the content provided, we provided a repeatable intervention, but this may have come at the cost of patient preferences for content and interests, and therefore immersion. There may also have been factors unique to the IUD procedure and our busy clinical environment that may have inhibited the formation of a therapeutic alliance.

Finally, due to the nature of this VR intervention, blinding of both clinicians and patients was not feasible.

### Future Directions

Future studies should be directed toward helping to improve the patient experience with IUD insertion. In addition to larger population studies to maximize generalizability of results and ability for subgroup analysis, there are several more specific areas identified by this study that warrant future consideration:

First, further qualitative studies regarding the other possible benefits of VR use for IUD insertion should be considered, given the positive response from patients regarding this despite the lack of improvement in pain and anxiety scores.

Second, the finding that preprocedural anxiety was most predictive of pain raises the question of whether providing anxiety management prior to IUD insertion could be beneficial. It may be that using VR prior to the procedure could be a useful way to reduce anxiety, potentially reduce pain, and ultimately improve procedural tolerability.

Finally, further research is required into optimizing VR content to maximize its interactivity and immersion, particularly in the gynecological context. Co-design with patients should be considered, and a balance will need to be reached between depth of VR immersion while also maintaining adequate situational awareness such that the patient can continue to provide consent to the intimate examinations being undertaken. This particularly requires cautious curation of the audio content to allow clinician communication with the patient. Visual imagery also needs to keep in mind the sensitive nature of this procedure type and therefore should avoid imagery that could be misconstrued or considered triggering for patients with a history of sexual trauma. This is a unique challenge that was felt to limit the appropriate options of VR content in this study and needs to be explored in depth in future VR research. Tailoring content for subgroup-specific features, such as heightened anxiety, or clinical characteristics, such as sexual trauma, may result in greater benefit.

### Conclusion

Importantly, this study found that the most significant predictor of pain was patient anxiety, consistent with Bennazouz et al [16] and the broader VR literature. This also predicted the patients who would benefit from VR use with reduced pain scores. This implies that efforts should be focused on managing patient anxiety prior to IUD insertion as a method of alleviating patient pain, and there may be a role for VR in the anxiety-reducing preprocedural setting that should be explored further in future research. The high satisfaction and recommendation for VR during IUD insertion in this trial also highlight that there may be other benefits to VR which have not yet been measured. This may indicate that VR could be considered as a supplemental tool rather than a primary method for pain relief. Ultimately, the goal to facilitate timely, more tolerable outpatient IUD insertions remains a clinical and research priority.

## Supplementary material

10.2196/72917 Multimedia Appendix 1 Survey provided to patients and clinicians for data collection.

10.2196/72917 Checklist 1 CONSORT Checklist.

## Abbreviations

IUD: intrauterine device
VAS: visual analog scale
VR: virtual reality
