# Supplementary material for: Virtual Reality for Analgesia During Intrauterine Device Insertion: Randomized Controlled Trial
Source: JMIR Serious Games. 2025 Sep 8;13:e72917. doi: 10.2196/72917 (PMC12416870; doi:10.2196/72917)
Supplement: Multimedia Appendix 1 [file games-v13-e72917-s001.pdf]

Date, time, researcher
Patient sticker

PATIENT

Before:
Patient reported anxiety

No anxiety — Anxiety as bad as it could be

During:

No pain — Pain as bad as it could be

No anxiety — Anxiety as bad as it could be

Have you used VR before?

1<br>Yes	2<br>No

How satisfied were you with the virtual reality headset?

1	2	3	4	5	6	7	8	9	10

How likely are you to recommend the virtual reality headset to a friend for IUD insertion?

1	2	3	4	5	6	7	8	9	10

Did you have any symptoms or complications from using the virtual reality headset?

Yes<br>List symptoms:	No

CLINICIAN

<u>Clinician evaluation:</u>

Clinician name:..

Clinician role:

1<br>Resident	2<br>Registrar	3<br>Consultant	4<br>Other

How easy was it to insert the mirena?

1<br>Easy	2<br>Very easy	3<br>Medium	4<br>Difficult	5<br>Very difficult

How many attempts were required to successfully insert the mirena?

1	2	3+	0<br>Unsuccessful insertion attempt

Time to insertion (min:sec)? ___:___
